# The Impact of Duodenal Diverticuli and the Execution of Endoscopic Retrograde Cholangiopancreaticography 

**DOI:** 10.1155/2016/5026289

**Published:** 2016-11-02

**Authors:** R. J. L. F. Loffeld, P. E. P. Dekkers

**Affiliations:** Department of Internal Medicine and Gastroenterology, Zaans Medisch Centrum, Zaandam, Netherlands

## Abstract

*Introduction.* Duodenal diverticuli alter the anatomy of the papillary region and can make an ERCP difficult.* Aim.* To study the outcome of ERCP in cases of duodenal diverticuli.* Patients and Methods.* Consecutive ERCPs in a period of 24 years were included. Endoscopy reports were studied for presence of diverticuli. Success of the procedure and findings were noted. Clinical records were searched for clinical presentation of the patient. Patients without duodenal diverticuli were used as comparison.* Results.* 2795 procedures were done in 2092 patients. Of these, 211 (10%) had diverticuli. Diverticuli occurred significantly more often in women (*p* < 0.001). ERCP was significantly more often inconclusive in cases of a diverticulum, 12.8% versus 6.3%, *p* < 0.001. In cases of a successful ERCP, patients with diverticuli showed more often no abnormalities in the bile duct, 26% versus 17%, *p* < 0.001. In 64% of cases, the reason for ERCP was cholestasis. There was no significant difference in presence of stones or cholangitis. Biliary pancreatitis was seen more often in patients without diverticuli, 4.4% versus 1.4%, *p* = 0.04. This was also the case for malignancies, 18.5% versus 6.6%, *p* < 0.001.* Conclusion.* It is concluded that duodenal diverticuli can be responsible for cholestasis. Presence of a diverticulum in the duodenum makes the ERCP procedure more complex.

## 1. Introduction

Diverticuli in the small intestine most often occur in the duodenum and are acquired during life. Specifically, periampullary diverticula are rare in patients below 40 years [1]. Although (periampullary) diverticuli usually do not cause symptoms, they can serve as a source of obstructive jaundice. This duodenal diverticulum obstructive jaundice syndrome is called Lemmel's syndrome [2]. In addition, the diverticulum alters the anatomy of the region of the papilla making identification of the papilla more difficult [3].

Periampullary diverticuli not only cause mechanical compression of the bile duct but also induce dysfunction of the sphincter of Oddi. They are considered to lead to bile stasis and to allow reflux from the duodenum into the bile duct, which can result in an ascending infection [4].

A close correlation between periampullary diverticuli and the formation of biliary tract stones has been reported. A particularly close correlation was found between diverticuli and stones in the common bile duct after cholecystectomy [5].

A study was done in consecutive patients undergoing endoscopic retrograde cholangiopancreatography (ERCP) for different clinical indications in order to study the clinical significance and outcome of the procedure in cases of duodenal diverticuli.

## 2. Patients and Methods

All consecutive ERCP procedures in a period of 24 years were included. All endoscopy reports were searched for patients with diverticuli in the duodenum. Success of the procedure as well as the findings was noted. Clinical records were searched for the indication and clinical presentation of the patient. Because many procedures were done a long time ago, only clinical records from patients undergoing the procedures in the last 13 years were included (these records were electronically available). As a control group, all patients undergoing ERCP without duodenal diverticuli were taken.

Statistical analysis was done with chi-square testing. A value below 0.05 was considered statistically significant.

## 3. Results

In the study period, 2795 procedures were done in 2092 patients. Of these, 211 (10%) had duodenal diverticuli.

Table 1 shows the diagnosis in patients with and without diverticuli. Diverticuli occurred significantly more often in women (*p* < 0.001).  Table 2 shows the clinical reason for the procedure in patients with diverticuli since 2003.

The procedure was significantly less often inconclusive in cases of absence of a diverticulum, 6.3% versus 12.8%, *p* < 0.001. On the other hand, in cases of a successful ERCP, patients with diverticuli showed significantly more often no abnormalities in the bile duct, 26% versus 17%, *p* < 0.001. There was no significant difference in presence of bile stones in the common bile duct nor was there a difference in the occurrence of cholangitis. However, biliary pancreatitis was seen more often in patients without diverticuli, 4.4% versus 1.4%, *p* = 0.04. This was also the case of malignancies, 18.5% versus 6.6%, *p* < 0.001.

## 4. Discussion

The prevalence of duodenal diverticuli, and more specifically periampullary diverticuli, shows a wide range. Periampullary diverticula are found in 9–32% of patients who undergo ERCP [1, 3]. Diverticuli in the papillary region can been seen in 5% of patients with calculous cholecystitis and in 9.5% of patients with choledocholithiasis. Patients with a postcholecystectomy syndrome have diverticuli in 30% of cases [6]. The size of the diverticula and position of the papilla in relation to the diverticula are variable. In a study, periampullary diverticuli were present in 7.5% of patients. Fifty-six percent of these were large and in the majority of cases (76%) the papilla was extradiverticular in location [7]. Boix et al. suggested a classification of periampullary diverticuli [8]. Unfortunately, in the present series, the exact relation of the diverticulum and the papilla was not always noted explicitly in the endoscopy reports.

Cannulation of the common bile duct is more difficult in patients with periampullary diverticulum and requires more skills and a longer learning curve [9, 10]. Nevertheless, it is stated that successful cannulation can be achieved. However, the effort and difficulty at attempting the goal of successful cannulation of the bile duct are different in patients with or without diverticuli [9]. In the present study, the number of inconclusive procedures, read as failed cannulation, was higher in cases of diverticuli. From the normal daily experience, it can be assumed that if cannulation failed, the papilla was located within a diverticulum beyond reach of the cannula.

In accordance with the literature, diverticuli in the duodenum were more seen in women [11].

It is reported in the literature that stones in the common bile duct occur significantly more often in patients with diverticuli [12, 13], indicating that the anatomical abnormality and malfunction of the sphincter of Oddi possibly play an important role in the formation of stones [14]. Also periampullary diverticuli are associated with larger stones and severe cholangitis [15, 16]. This could not be confirmed in the present study; stones in the common bile duct as well as cholangitis occurred in both groups of patients.

It could be assumed that, due to the changed anatomy, complications would occur more often in cases of a papilla in or in close vicinity of a diverticulum. According to the literature, this is not the case. ERCP appeared to be a safe procedure also in patients with diverticuli, with a good success rate and low complications [1, 10, 13], although there is one study reporting more post-ERCP pancreatitis [11].

It is feasible to suggest that periampullary diverticuli deteriorate the evacuative function of the bile duct due to compression of terminal parts of the choledochus [6]. In the present study, the common bile duct often was normal in the presence of a diverticulum. The majority of patients underwent the ERCP because of presence of painful cholestasis. This is an indirect argument for food impaction in the diverticulum causing complaints and cholestasis. Indeed, diverticuli in the duodenum can contain food residue, ultimately leading to obstruction of the bile outflow tract with jaundice. A food bezoar can be misdiagnosed as a tumour located at the papillary region [17].

Cancer was more often diagnosed in patients without diverticuli. The meaning of this observation is not obvious. In fact, it is a bridge too far to suggest that diverticuli in the duodenum are protective against the development of cancer.

It is concluded that presence of diverticuli in the periampullary region is an extra challenge for performing ERCP. Food impaction probably is far more responsible than bile stones for obstruction of the outflow tract leading to pain and cholestasis.

## Figures and Tables

**Table 1:** Findings during ERCP in patients with or without diverticuli. More than one diagnosis can be present in a single patient. The number between brackets is the percentage.

	With diverticuli	Without diverticuli
Men	66 (31.3)	769 (40.9)
Women	145 (68.7)	1112 (59.1)
gallstones	109 (51.7)	932 (49.5)
No abnormalities	55 (26)	320 (17)
Cholangitis	9 (4.2)	66 (3.5)
Pancreatitis	3 (1.4)	82 (4.4)
Cancer	14 (6.6)	348 (18.5)
Inconclusive	27 (12.7)	119 (6.3)

**Table 2:** Clinical reasons for performing the ERCP in patients with duodenal diverticuli in 94 patients with available clinical records.

Painful cholestasis	60
Jaundice	8
Nonpainful cholestasis	1
Cholangitis	13
Pancreatitis	4
Dilated bile ducts	7
Unknown	1
